# Room for Two: The Synaptophysin/Synaptobrevin Complex

**DOI:** 10.3389/fnsyn.2021.740318

**Published:** 2021-09-20

**Authors:** Dustin N. White, Michael H. B. Stowell

**Affiliations:** MCD Biology, University of Colorado Boulder, Boulder, CO, United States

**Keywords:** synaptic fusion, fusion machinery, supercomplex, synaptobrevin, synaptophysin (SYP)

## Abstract

Synaptic vesicle release is regulated by upwards of 30 proteins at the fusion complex alone, but disruptions in any one of these components can have devastating consequences for neuronal communication. Aberrant molecular responses to calcium signaling at the pre-synaptic terminal dramatically affect vesicle trafficking, docking, fusion, and release. At the organismal level, this is reflected in disorders such as epilepsy, depression, and neurodegeneration. Among the myriad pre-synaptic proteins, perhaps the most functionally mysterious is synaptophysin (SYP). On its own, this vesicular transmembrane protein has been proposed to function as a calcium sensor, a cholesterol-binding protein, and to form ion channels across the phospholipid bilayer. The downstream effects of these functions are largely unknown. The physiological relevance of SYP is readily apparent in its interaction with synaptobrevin (VAMP2), an integral element of the neuronal SNARE complex. SNAREs, soluble NSF attachment protein receptors, comprise a family of proteins essential for vesicle fusion. The complex formed by SYP and VAMP2 is thought to be involved in both trafficking to the pre-synaptic membrane as well as regulation of SNARE complex formation. Recent structural observations specifically implicate the SYP/VAMP2 complex in anchoring the SNARE assembly at the pre-synaptic membrane prior to vesicle fusion. Thus, the SYP/VAMP2 complex appears vital to the form and function of neuronal exocytotic machinery.

## Introduction

**Figure 1:**
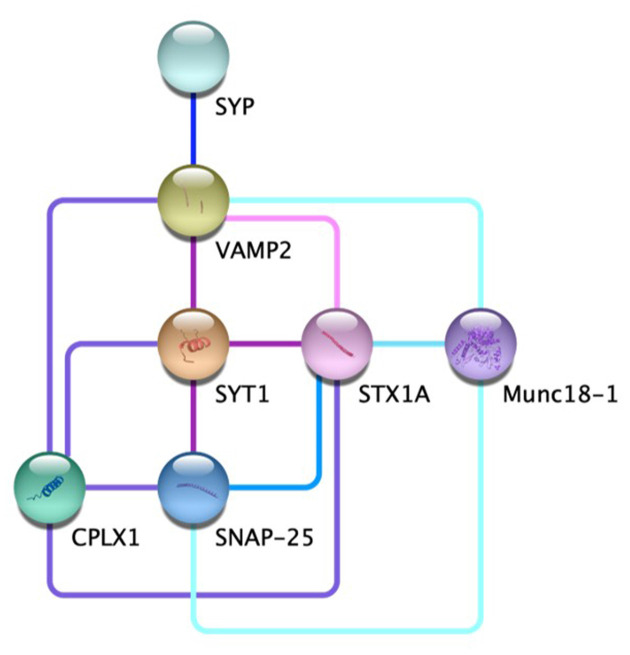
The protein-protein interaction network at the chemical synapse. Direct evidence for the edges displayed for VAMP2-STX1A 
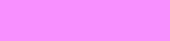
 (Far et al., 1995; Hazzard et al., 1999); VAMP2-SYT1-SNAP25 
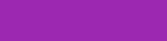
 (Söllner et al., 1993a,b; Chapman et al., 1995; Zhang et al., 2002); VAMP2-SYT1- SYTX1A-CPLX1 
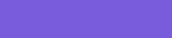
 (Pabst et al., 2000; Bracher et al., 2002; Chen et al., 2002; Zhou et al., 2017); SYP-VAMP2 
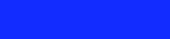
 (Edelmann et al., 1995; Felkl and Leube, 2008; Gordon et al., 2011; Adams et al., 2015); STX1A-SNAP25 
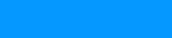
 (McMahon and Südhof, 1995; Fasshauer et al., 1997); STX1A-Munc18-1 
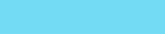
 (Hata et al., 1993; Misura et al., 2000; Zilly et al., 2006; Rickman et al., 2007); VAMP2-SNAP25-Munc18-1 
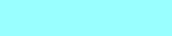
 (Carr et al., 1999; Dulubova et al., 2007).

Communication between neurons is a fundamental process of the nervous system. Regulated neurotransmitter release contributes to everything from memory consolidation to mood regulation (Jurado et al., 2013; Kandel et al., 2014; Metzger et al., 2017). Aberrant synaptic release is associated with numerous neurological disorders, and the molecular mechanisms underlying this process are elaborate (Roselli and Caroni, 2015; Körber and Kuner, 2016; Ramos-Miguel et al., 2018). The general role of vesicle and target SNAREs, v-SNAREs and t-SNAREs respectively, in vesicle fusion at the pre-synaptic membrane has been widely studied, but some of the individual components of this pathway are more nebulous (Karmakar et al., 2019). Specifically, SYP, while prolific at most pre-synaptic terminals, has no well-defined role within the synaptic architecture (Marqueze-Pouey et al., 1991). Putative functions of the vesicle membrane protein SYP include vesicular ion channel activity, vesicle endocytosis, synaptobrevin trafficking during SNARE assembly, and the kiss-and-run archetype of dense-core vesicle fusion (Gincel and Shoshan-Barmatz, 2002; Kwon and Chapman, 2011; Harper et al., 2017; Chang et al., 2021). Probing these functions has proved difficult, however, due to the compensatory nature of various physin family proteins (Janz et al., 1999). SYP’s interaction with VAMP2 is of particular interest; the two proteins form a complex thought to contribute to the characteristic speed and reactivity of synaptic vesicle release (Adams et al., 2015). Recent advances have further illuminated the individual and cooperative roles of SYP and VAMP2 in synaptic vesicle regulation. Recent advances have further illuminated the individual and cooperative roles of SYP and VAMP2 in synaptic vesicle regulation as well as the interaction network between other key players in vesicle fusion (Figure 1, Table 1; Szklarczyk et al., 2019).

**Table 1:** Network evidence—STRING false discovery rate (FDR) < 1e-10.

Article title	Author	FDR
Candidate pathway association study in cocaine dependence: the control of neurotransmitter release	Fernàndez-castillo et al. (2012)	1.89E-15
Subtle Interplay between synaptotagmin and complexin binding to the SNARE complex	Xu et al. (2013b)	1.47E-13
Munc18a does not alter fusion rates mediated by neuronal SNAREs, synaptotagmin, and complexin	Zhang et al. (2002)	8.50E-13
Quantitative Proteomic Analysis Reveals Molecular Adaptations in the Hippocampal Synaptic Active Zone of Chronic Mild Stress-Unsusceptible Rats.	Zhou et al. (2015)	9.85E-13
Solution NMR of SNAREs, complexin, and α-synuclein in association with membrane-mimetics	Liang and Tamm (2018)	9.85E-13
MicroRNA-153 impairs presynaptic plasticity by blocking vesicle release following chronic brain hypoperfusion	Yan et al. (2020)	3.15E-11
Ca^2+^-Triggered Synaptic Vesicle Fusion Initiated by Release of Inhibition	Brunger et al. (2018)	7.38E-11
*De novo* STXBP1 mutations in mental retardation and nonsyndromic epilepsy	Hamdan et al. (2009)	9.81E-11
The cell adhesion protein CAR is a negative regulator of synaptic transmission	Wrackmeyer et al. (2019)	2.15E-10
Identification of SNARE and cell trafficking regulatory proteins in the salivary glands of the lone star tick, Amblyomma americanum (L.)	Karim et al. (2002)	2.47E-10
Extended Synaptotagmin (ESyt) Triple Knock-Out Mice Are Viable and Fertile without Obvious Endoplasmic Reticulum Dysfunction	Sclip et al. (2016)	2.71E-10
Impaired gene and protein expression of exocytotic soluble N-ethylmaleimide attachment protein receptor complex proteins in pancreatic islets of type 2 diabetic patients.	Ostenson et al. (2006)	3.34E-10
Munc18-1 binding to the neuronal SNARE complex controls synaptic vesicle priming	Deák et al. (2009)	3.34E-10
Munc13 mediates the transition from the closed syntaxin-Munc18 complex to the SNARE complex	Ma et al. (2011)	3.34E-10
The synaptic pathology of cognitive life	Honer et al. (2019)	3.34E-10
A single amino acid mutation in SNAP-25 induces anxiety-related behavior in mouse	Kataoka et al. (2011)	3.90E-10
Re-examining how complexin inhibits neurotransmitter release	Trimbuch et al. (2014)	3.90E-10
Components of the neuronal exocytotic machinery in the anterior pituitary of the ovariectomized ewe and the effects of estrogen in gonadotropes as studied with confocal microscopy.	Thomas et al. (1998)	3.90E-10
The Janus-faced nature of the C(2)B domain is fundamental for synaptotagmin-1 function	Xue et al. (2008)	4.92E-10
Mutations in the Neuronal Vesicular SNARE VAMP2 Affect Synaptic Membrane Fusion and Impair Human Neurodevelopment	Salpietro et al. (2019)	4.92E-10
Munc18-1 is crucial to overcome the inhibition of synaptic vesicle fusion by αSNAP	Stepien et al. (2019)	4.92E-10
GPCR regulation of secretion	Yim et al. (2018)	8.02E-10

### Synaptophysin

Despite its prevalence at the pre-synaptic terminal, synaptophysin’s role in vesicular neurotransmission is highly speculative. Synaptophysin (SYP) forms a transmembrane structure on synaptic vesicles similar to canonical gap junctions and mechanosensitive ion channels (Arthur and Stowell, 2007). This structure is concordant with the idea that SYP forms functional ion channels within membranes (Yin et al., 2002). Indeed, SYP multimers reconstituted in phospholipid bilayers are selective for cations and show a preference for potassium (Gincel and Shoshan-Barmatz, 2002). Though past studies saw no change in ion channel activity in response to fluctuation in Ca^2+^ concentration, SYP is known to bind cytoplasmic calcium (Rehm et al., 1986). The importance of calcium-dependent exocytosis in neurotransmitter release is widely accepted, but the specific function of SYP-Ca^2+^ binding is unclear (Karmakar et al., 2019).

In addition to its association with Ca^2+^, SYP readily binds cholesterol in the plasma membrane. This binding is necessary for the initial formation of synaptic vesicles (Thiele et al., 2000). Further roles for cholesterol in the function of SYP, including regulation of synaptic plasticity and the interaction of synaptophysin with synaptobrevin, have been suggested, but the precise physiology underlying this relationship remains elusive (Mitter et al., 2003; Ya et al., 2013).

### Synaptobrevin

VAMP2, syntaxin, and SNAP-25 form the core assembly of SNARE proteins (Brunger, 2005). The collaborative functions of these and other SNARE-associated proteins are necessary for Ca^2+^-dependent neurotransmitter release at presynaptic terminals (Weber et al., 1998). VAMP2 knockout mice exhibit profoundly reduced rates of vesicle fusion, though fusion is not abrogated entirely (Schoch et al., 2001). While VAMP2 alone may not be necessary for fusion overall, its absence profoundly affects the rate of neurotransmission. Additionally, VAMP2/VAMP3 double knockout results in complete termination of presynaptic vesicle fusion, reinforcing the significance of synaptobrevin and its structural homologs (Borisovska et al., 2005).

VAMP2 may also be required for the maintenance of the readily releasable pool (RRP). Specifically, VAMP2 appears to be associated with the “fast endocytosis” necessary for quick Ca^2+^ signaling. At terminals with depleted RRPs, the rate of vesicle recycling is significantly impacted by the absence of VAMP2 (Deák et al., 2004). “Slow endocytosis” is also VAMP2 dependent, but appears to rely on the activity of additional SNAREs syntaxin and SNAP-25 (Xu et al., 2013a). This dual role of VAMP2 heavily implicates it in the cycle of exo- and endocytosis required to maintain the RRP.

### SYP/VAMP2 Complex

#### Exocytosis

While Ca^2+^ induced exocytosis is widely recognized as the basis of neurotransmitter release, the molecular architecture underlying this process is a point of contention (Berridge, 1998; Neher and Sakaba, 2008; Williams and Smith, 2018). The neuronal SNARE fusion complex at the pre-synaptic plasma membrane results from the assembly of numerous fusion proteins, including syntaxin, SNAP-25, and synaptobrevin (VAMP2; Brunger, 2005). Recent cryoelectron tomographic evidence points to a conserved, symmetric distribution of proteins at primed active zones. While the identity of these proteins is unknown, it is clear that their assembly is dependent on the upstream function of SNARE and SNARE-interacting molecules (Radhakrishnan et al., 2021).

One such complex is formed by SYP and VAMP2. This hexameric complex is thought to provide a template for the assembly of proteins at primed active zones, thereby controlling exocytosis through regulation of VAMP2 binding (Edelmann et al., 1995). The SYP/VAMP2 complex, assembled prior to docking and priming, is functionally suited for this purpose: the early assembly of SYP/VAMP2 facilitates a quick temporal response necessary for Ca^2+^-mediated exocytosis (Adams et al., 2015). Interestingly, there is evidence that SYP is specifically involved in the *negative* regulation of VAMP2-syntaxin binding, further supporting the importance of synaptophysin in temporal control of SNARE formation (Raja et al., 2019). SYP mutations that affect VAMP2 trafficking are associated with neurodevelopmental disorders, and understanding the molecular sequence of events underlying SYP/VAMP2-mediated trafficking is vital for addressing public health concerns (Harper et al., 2017; John et al., 2021).

#### Endocytosis

In addition to neurotransmitter release, vesicular recycling at the synaptic cleft is also thought to be influenced by the SYP/VAMP2 complex. Synaptophysin itself is crucial to the maintenance of synaptic vesicle endocytosis; SYP loss-of-function mutations result in severely reduced recycling rates (Kwon and Chapman, 2011). During vesicle recycling, SYP appears to be required for the reuptake of VAMP2 into the pre-synaptic bouton, as well as maintaining appropriate levels of VAMP2 at the pre-synapse (Gordon et al., 2011; Kokotos et al., 2019). Additionally, this relationship is heavily reliant upon the ratio of SYP to VAMP2; disruptions in the physiological balance of the two proteins results in drastically reduced trafficking of VAMP2 back to pre-synaptic vesicles (Gordon et al., 2016).

Endocytosis is primarily dependent on SYP’s cytoplasmic C-terminus, which is necessary for the efficient recovery of VAMP2 (Harper et al., 2021). While the direct molecular basis of SYP/VAMP2 binding is becoming clearer, there are several extrinsic factors that contribute to the complex’s role in overall synaptic function. A particularly interesting property of the SYP/VAMP2 complex is its dependence on cholesterol, with assembly preferentially occurring in high cholesterol environments (Mitter et al., 2003; Hussain et al., 2019). With further investigation, external components like cholesterol may reveal functional links to some puzzling physiologies. One example is the predilection of synapses for recently synthesized vesicle proteins. Synaptophysin exits the recycling pool relatively quickly following production and is replaced by freshly-synthesized molecules; how this ties into the balance of SYP/VAMP2 is unknown (Truckenbrodt et al., 2018).

### Contextual Roles

Many integral pre-synaptic functions remain ambiguously defined at the molecular level. The calcium ion is a key player in vesicular docking and fusion; Ca^2+^ concentration is directly related to the rate of vesicle recruitment and release (Neher and Sakaba, 2008). The direct role of Ca^2+^ within the pre-synaptic terminal is highly contextual and depends on local signal strength as well as the type of synapse (excitatory vs. inhibitory; Schneggenburger and Neher, 2005; Williams and Smith, 2018). Despite this physiological range, many Ca^2+^ sensors have been identified at the pre-synaptic terminal. Regulation of the readily releasable pool (RRP) is one process that relies upon such Ca^2+^ sensors. The RRP links Ca^2+^ flux to synaptic strength and release probability (Thanawala and Regehr, 2013). In addition, Munc18-1 has been established as a key physiological regulator of cognate SNARE activation (Shen et al., 2007). Munc18-1 contributes to the maintenance of the SNARE complex in the presence of destabilizing factors. This kind of synapse-specific Ca^2+^ response could be important in the molecular regulation of atypical synaptic release.

In addition to traditional synapses, which use a single primary neurotransmitter, so-called dual-release terminals are found in many pathways throughout the brain and are capable of producing and releasing two primary neurotransmitters, such as glutamate and GABA (Vaaga et al., 2014; Root et al., 2018). These terminals release GABA and glutamate at a specific ratio, and disruptions of this ratio are implicated in major depressive disorder and addiction (Shabel et al., 2014; Root et al., 2020). Despite the prevalence and associated pathologies of dual-release neurons, the pre-synaptic machinery regulating most release properties is unknown.

**Figure 2:**
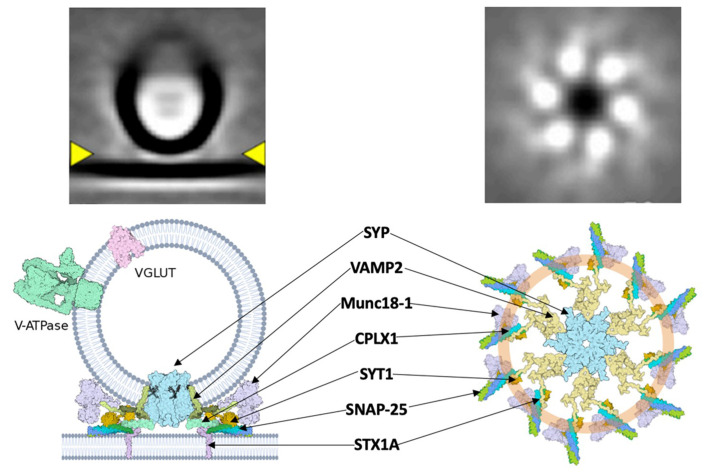
A hypothetical synaptic fusion nanomachine comprised of a multimeric assembly organized by synaptophysin. **Top**, cryo-ET averages of docked synaptic vesicles (Radhakrishnan et al., 2021). **Bottom**, proposed fusion assembly based upon the prior synaptophysin/synaptobrevin complex (Adams et al., 2015) and the buttressed ring model (Rothman et al., 2017).

A major connection between dual-release physiology and Ca^2+^-mediated vesicular release may be found in the SYP/VAMP2 complex and associated superstructures. A central Ca^2+^ sensor, synaptotagmin (syt), is responsible for the induction of synchronous events at the pre-synaptic membrane through regulation of vSNARE activity *via* its C2B domain (Chang et al., 2018). Syt has recently been shown to form a ring-like structure upon which SNAREs are assembled (Rothman et al., 2017). Specifically, the C2AB domain of syt “clamps” the SNARE assembly at the synaptic vesicle (Figure 2). This conceptually allows docking of the vesicle at the plasma membrane with the syt ring attached, followed by Ca^2+^-mediated disassembly of the syt ring upon action potential stimulation and subsequent fusion of vSNAREs to the membrane. This framework could contribute to the previously described speed of SNARE complex assembly, vesicle docking, and fusion. The physiological role of syt extends beyond a simple scaffold, as demonstrated in its ability to negatively regulate the attachment of vSNAREs to their plasma membrane-bound tSNARE targets at low intracellular Ca^2+^ concentrations (Wang et al., 2014). Interestingly, it appears that the syt “ring” acts as a vesicle “landing gear,” guiding a pre-synaptic vesicle to specific active zones determined by the concentration of PIP2 (Zhu et al., 2021). The specificity of this docking might elucidate the basis of ratio-based dual-release. If the two neurotransmitters of a dual-release terminal are packaged in separate vesicles, an idea that has recently been contested, then one of the logical methods for regulating the ratio of release is *via* differential trafficking to the pre-synaptic membrane (Wang et al., 2020; Kim et al., 2021). The interaction of syt with the vesicular SYP/VAMP2 complex provides a structural basis for distinct trafficking pathways, but further study is needed to define the precise mechanism. Understanding how the v-SNARE pantheon contributes to dual-release physiology is essential for refining signaling pathways, understanding mood regulation, and discovering targeted treatments for associated disorders. The key to this understanding will come by obtaining high-resolution structure data of the SYP/VAMP complex so that mutational disruption of the complex can be explored functionally. Methods such as cryo-electron tomography and super-resolution microscopy will be vital next steps in analyzing pre-synaptic structures at a sufficient resolution. Questions regarding the spatial arrangement, temporal dynamics, and association kinetics of SNARE-associated complexes are crucial yet remain unanswered.

## Author Contributions

DW and MS wrote the manuscript and approved it for publication. All authors contributed to the article and approved the submitted version.

## Conflict of Interest

The authors declare that the research was conducted in the absence of any commercial or financial relationships that could be construed as a potential conflict of interest.

## Publisher’s Note

All claims expressed in this article are solely those of the authors and do not necessarily represent those of their affiliated organizations, or those of the publisher, the editors and the reviewers. Any product that may be evaluated in this article, or claim that may be made by its manufacturer, is not guaranteed or endorsed by the publisher.
